# Aging interferes central control mechanism for eccentric muscle contraction

**DOI:** 10.3389/fnagi.2014.00086

**Published:** 2014-05-09

**Authors:** Wan X. Yao, Jinqi Li, Zhiguo Jiang, Jia-Hong Gao, Crystal G. Franklin, Yufei Huang, Jack L. Lancaster, Guang H. Yue

**Affiliations:** ^1^Department of Kinesiology, Health, and Nutrition, College of Education and Human Development, The University of Texas at San AntonioSan Antonio, TX, USA; ^2^Research Imaging Institute, The University of Texas Health Science Center at San AntonioSan Antonio, TX, USA; ^3^Human Performance and Engineering Laboratory, Kessler Foundation Research CenterWest Orange, NJ, USA; ^4^Department of Biomedical Engineering, New Jersey Institute of TechnologyNewark, NJ, USA; ^5^IDG/McGovern Institute for Brain Research, Peking UniversityBeijing, China

**Keywords:** brain activation, concentric contraction, eccentric contraction, fMRI, movement stability

## Abstract

Previous studies report greater activation in the cortical motor network in controlling eccentric contraction (EC) than concentric contraction (CC) despite lower muscle activation level associated with EC vs. CC in healthy, young individuals. It is unknown, however, whether elderly people exhibiting increased difficulties in performing EC than CC possess this unique cortical control mechanism for EC movements. To address this question, we examined functional magnetic resonance imaging (fMRI) data acquired during EC and CC of the first dorsal interosseous (FDI) muscle in 11 young (20–32 years) and 9 old (67–73 years) individuals. During the fMRI experiment, all subjects performed 20 CC and 20 EC of the right FDI with the same angular distance and velocity. The major findings from the behavioral and fMRI data analysis were that (1) movement stability was poorer in EC than CC in the old but not the young group; (2) similar to previous electrophysiological and fMRI reports, the EC resulted in significantly stronger activation in the motor control network consisting of primary, secondary and association motor cortices than CC in the young and old groups; (3) the biased stronger activation towards EC was significantly greater in the old than the young group especially in the secondary and association cortices such as supplementary and premotor motor areas and anterior cingulate cortex; and (4) in the primary motor and sensory cortices, the biased activation towards EC was significantly greater in the young than the old group. Greater activation in higher-order cortical fields for controlling EC movement by elderly adults may reflect activities in these regions to compensate for aging-related impairments in the ability to control complex EC movements. Our finding is useful for potentially guiding the development of targeted therapies to counteract age-related movement deficits and to prevent injury.

## INTRODUCTION

Our daily movements consist of shortening (concentric) and lengthening (eccentric) muscle contractions and it has well been documented that mechanical and neurophysiological characteristics of an eccentric contraction (EC) differ in many ways from those of a concentric contraction (CC) despite the fact that the two types of muscle activities are accomplished by the same muscle(s; See Duchateau and Enoka, 2008 for review). For example, after a period (30 min) of repetitive eccentric and concentric contractions at 50% maximal intensity, only those who performed eccentric exercise showed a significant reduction in mechanical stiffness and an increase in magnetic resonance imaging (MRI) T_2_ relaxation time of the working muscle (Sesto et al., 2008). In addition, previous studies (Nardone et al., 1988; Howell et al., 1995) suggest that EC and CC may follow different motor-unit recruitment orders during non-fatigue muscle contractions [However, see Bawa and Jones (1999) and Kossev and Christova (1998) for contradictory findings]. Furthermore, compared to CC, EC had a smaller magnitude of electromyographic (EMG) signal against a given resistance, and depressed corticospinal neuron (Abbruzzese et al., 1994; Sekiguchi et al., 2001) and monosynaptic reflex (Moritani et al., 1988; Abbruzzese et al., 1994) excitability. Moritani et al. (1988) attributed the lower level of EMG for EC to fewer motor units being recruited and a lower discharge rate of the active motor units. Recent studies by Duclay and Martin (2005), Duclay et al. (2011) postulate that the differences in EMG activities between the two types of contractions are a result of differential modulations of motoneuron excitability at supraspinal and/or spinal levels and the modulation of the spinal motoneuron excitability by the supraspinal centers can be contraction-type specific (Duclay et al., 2009). T together, the aforementioned findings indicate a possible different neural control strategy for EC from the one for CC.

In an attempt to delineate potential differential central control mechanisms between voluntary EC and CC, Fang et al. (2001, 2004)conducted two studies to monitor electroencephalography (EEG) signals at submaxial and maximal intensity levels. They found that although the elbow flexor muscle activities (EMG) were lower during EC than CC, the magnitude of movement-related cortical potential (MRCP) derived from the EEG recordings was significantly greater for EC than CC at both intensity levels. They hypothesized that the greater cortical signal (MRCP) for EC might be due to greater effort in planning and programming the lengthening contractions that are more difficult to perform, prone to muscle tissue damage, and may possibly be involved with a different control strategy such as a reversed motor unit recruitment order. More recently, Kwon and Park (2011) examined brain activation patterns by quantifying functional MRI (fMRI) signals in the primary motor cortex, inferior parietal lobe, pre-supplementary area, anterior cingulate cortex, prefrontal cortex, and cerebellum in healthy young adults. They found that except in the right primary motor cortex, the EC resulted in greater fMRI signals than CC in all examined cortical areas. In summary, all three studies that directly monitored brain activities (Fang et al., 2001, 2004; Kwon and Park, 2011) reported a greater activation level in the cortical functional neural network in controlling eccentric than concentric muscle contractions despite a lower muscle activation level associated with EC than CC in healthy young individuals.

In the field of motor control research in aging, literature has consistently demonstrated that elderly individuals exhibit poorer movement stability or force steadiness during EC than CC (Laidlaw et al., 1999, 2002). Although movement stability during EC is also poorer than CC in young individuals, the magnitude of deficit is significantly larger in old than young age groups (Laidlaw et al., 1999, 2002), indicating force/movement control ability for EC is more affected in older adults and this poses greater risks for injury during EC activities. However, central control mechanisms between CC and EC in older adults have never been investigated. Given the expectation that poor control of EC by muscles such as quadriceps during walking downstairs may increase chances of falls, it is important to understand the central nervous systems (CNS) physiological factors contributing to a loss of EC control ability in later life. All findings from previous research seem to support the notion that differential central control mechanisms for EC and CC movements reflect activities of the CNS in dealing with unique strategies in recruiting motor units, altered means in producing force and power, and increased risks of tissue damage during EC. However, it is unknown whether old people exhibiting increased difficulties in performing EC than CC still possess the unique cortical control mechanism for EC like their younger counterparts. The purpose of the study was to address this question. It was hypothesized that the control mechanism (indicated by brain activation pattern) for EC and CC in an aging population would be different from the one adopted by young individuals based on the observation of different neuromuscular characteristics in aging including substantially worsened EC performance compared to young adults (Burnett et al., 2000; Hortobagyi et al., 2001; Laidlaw et al., 2002).

## MATERIALS AND METHODS

### SUBJECTS

Eleven young (4 males, 23.25 ± 4.09 years old, ranging from 20 to 32 years) and nine old (3 males, 68.72 ± 3.14 years old, ranging from 67 to 73) subjects participated in the study. All subjects were right-handed determined by the Edinburgh inventory (Oldfield, 1971) without neurological, neuromuscular, and musculoskeletal impairments. The study was approved by the Institutional Review Boards of the University of Texas at San Antonio and the University of Texas Health Science Center at San Antonio. Written informed consents were obtained from all the subjects prior to their participation.

### TASKS AND EXPERIMENTAL SETUP

The subjects performed either concentric (muscle shortening, index finger moving towards the thumb) or eccentric (muscle lengthening, index finger passively moving away from the thumb) contractions of the first dorsal interosseous (FDI) muscle. Each type of contraction resulted in a 20-degree angular movement from the initial position against a constant load (30% of maximal isometric contraction of the FDI) with a constant speed (~10 degree/s or ~0.174 rad/s). After a practicing block of 20 concentric contractions (CC) and 20 eccentric contractions (EC), a total of 20 CC and 20 EC testing trials were performed while functional brain images were taken. During the practice and testing, CC and EC were performed alternately (e.g., CC → EC → CC…) while the subjects were lying supine in the MRI chamber with right arm and hand resting prone on a wooden board. The right arm was abducted 10° at shoulder joint with the elbow joint flexed to ~10°. The wrist, thumb, and the other three (middle, ring, and little) fingers of the right hand were constrained. The right index finger was fastened to a movable lever attached to a load (30% maximal) through a non-elastic cable and a pulley fixed on the wooden board. The subjects performed CC of the right FDI muscle from the initial position (IP) by lifting the weight to the end position (EP) and EC from the EP by “lowering” the weight to the IP. A 10-s rest was provided after each contraction during which the weight was supported by an external mechanism.

### MEASUREMENTS AND DATA PROCESSING

#### Maximum voluntary contraction

Isometric maximum voluntary contraction (MVC) force was measured by requesting the subjects maximally abduct their right index figure against an unmovable force transducer (Sensotec, Columbus, OH, USA). Each subject performed two MVC trials and the trial with the higher peak force was chosen for further analysis. There was a 1-min rest between the two MVC trials.

#### Movement distance, speed and stability

A custom-built, MRI-compatible goniometer for measuring the angular/movement distance and speed of the finger was attached to the movable lever. The movement data for both CC and EC were trigger-averaged across the 20 trials for each subject with a trigger signal, which was generated when the finger moved 2° from the IP (10% of the movement distance) for CC and 2° from the EP (Fang et al., 2001, 2004). The movement speed was then derived from the trigger-averaged movement data. Standard deviation of the speed was taken to indicate stability of each movement.

#### Image acquisition

The fMRI procedures (Gao et al., 1996; Yue et al., 2000) are based on detecting changes in brain blood oxygenation – the so-called blood oxygenation level dependent (BOLD) signal changes. Functional images were acquired by a 3-T Magnetom Trio scanner (Siemens, Erlangen, Germany) with a 8-channel RF head coil using a single shot gradient echo EPI pulse sequence (TR = 2 s, TE = 30 ms, flip angle = 90°). The subjects were positioned supine on the sliding board of the scanner with his/her head positioned in the head coil. The head was stabilized by padded restraints. Subjects were told to remain as still as possible during the experiments. Both T1-weighted anatomical images and functional images were collected in the same transverse plane (aligned with a line connecting the anterior and posterior commissures). Each brain volume consisted of 21 slices (5 mm slice thickness and 1 mm inter slice gap) that covered the brain and cerebellum. (The collection of 1 brain volume was referred to as 1 scan hereafter.) The image field of view and matrix for the anatomical images were 256 × 256 mm and 256 × 256, respectively; and those for the function images were 256 × 256 mm and 128 × 128, yielding an in-plane spatial resolution of 1 × 1 mm for anatomical images and 2 × 2 mm for functional images. Pulse sequence for the anatomic image acquisition was TR/TE/flip angle = 2200 ms/2.83 ms/13°, non-selective inversion pulse, TI = 785 ms. All anatomical images were acquired after fMRI scans.

### DATA ANALYSIS

The fMRI data were processed using FMRIB Software Library (FSL; Woolrich et al., 2009) and codes written in MATLAB (Math Works, Natick, MA). Image pre-processing procedures included skull stripping, motion correction, temporal filtering, and spatial smoothing with a Gaussian filter of 5 mm at FWHM. A non-linear Gaussian temporal filter was applied using a high-pass threshold of 100 s. Activation maps were generated using the general linear modeling (GLM) in the FEAT toolbox in FSL on a voxel-by-voxel basis and a threshold of *Z* = 3.0 (*p* < 0.01). All echo-planar images were co-registered first to each subject’s corresponding anatomical images and then to the Collin brain, a high-resolution template (0.2 mm isotropic resolution) from a population-based, pseudo-Talairach space, before the activated volume and the subsequent group fMRI data were analyzed. A 2-way ANOVA (group × task) was employed to analyze movement parameters (speed and stability) and fMRI (volume passing activation threshold) results in each brain region showing CC- or EC-related activation. To better understand the relative activation level in each cortical area displaying strong activities during CC and EC, we calculated an EC-to-CC activation ratio in each area in each group. Independent *t*-test was employed to analyze the ratio data between the groups. Statistic significance level was set at *P* = 0.05 for all the analyses.

## RESULTS

### MVC

The mean MVC forces for the young and old groups were 48.9 ± 3.67 N and 40.3 ± 3.89 N, respectively. The independent *t*-test revealed a non-significant difference in MVC force between the two groups, *t*(18) = 1.597, *p* = 0.128.

### MOVEMENT SPEED AND STABILITY

**FIGURE 1:**
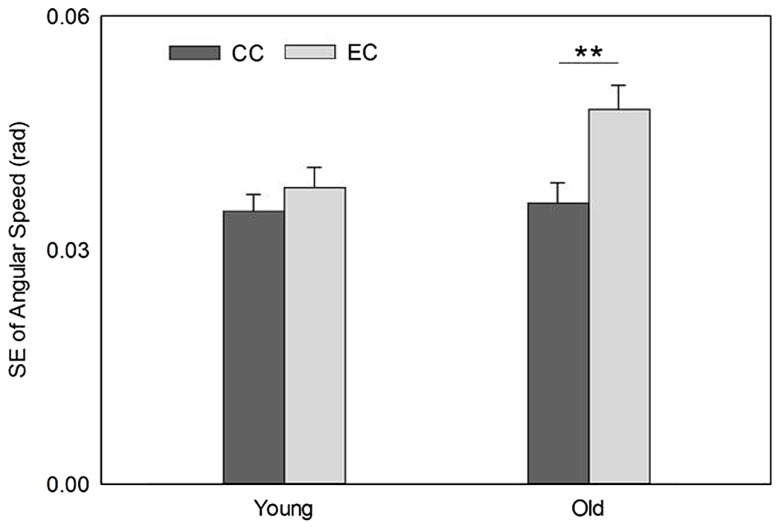
**Movement stability was poorer during EC (indicated by greater standard error [SE]) than CC in the old group but no such difference in the young group.** **P < 0.01.

The mean speeds of EC and CC for the young group were 0.167 ± 0.015 rad/s and 0.174 ± 0.011 rad/s, respectively. For the old group, the speeds of EC and CC were 0.171 ± 0.020 rad/s and 0.170 ± 0.014 rad/s, respectively. The main effects for the age group and movement type analyzed by the 2-way ANOVA were not significant, *F*(1,18) = 0.003, *P* = 0.96, and *F*(1,18) = 0.252, *P* = 0.622, respectively; nor was the interaction between the two factors, *F*(1,18) = 0.51, *P* = 0.484. The standard deviations of the speed, representing movement stability, revealed a significant main effect for movement type but not group, *F*(1,18) = 15.65, *P* = 0.001, and *F*(1,18) = 2.572, *P* = 0.126, respectively. The interaction between the two factors on the movement stability was also significant, *F*(1,18) = 5.896, *P* = 0.026. The *post hoc* paired *t*-test on the movement stability was not significant between EC and CC for the young group, *t*(10) = 1.299, *P* = 0.22. In contrast; it was significant between the two movement types for the old group, *t*(8) = 3.789, *P* < 0.01 (**Figure 1**).

### fMRI

**FIGURE 2:**
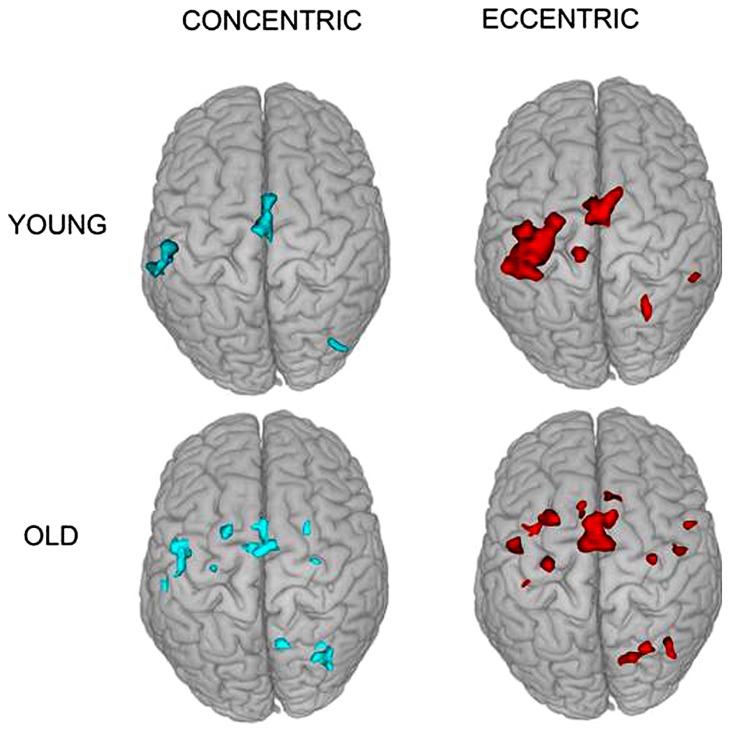
**Superior view of group activation during the two types of contractions in the two groups.** It is clear that brain activation in young individuals are more localized in the contralateral (left) hemisphere but that in older adults are more distributed in the two hemispheres.

**FIGURE 3:**
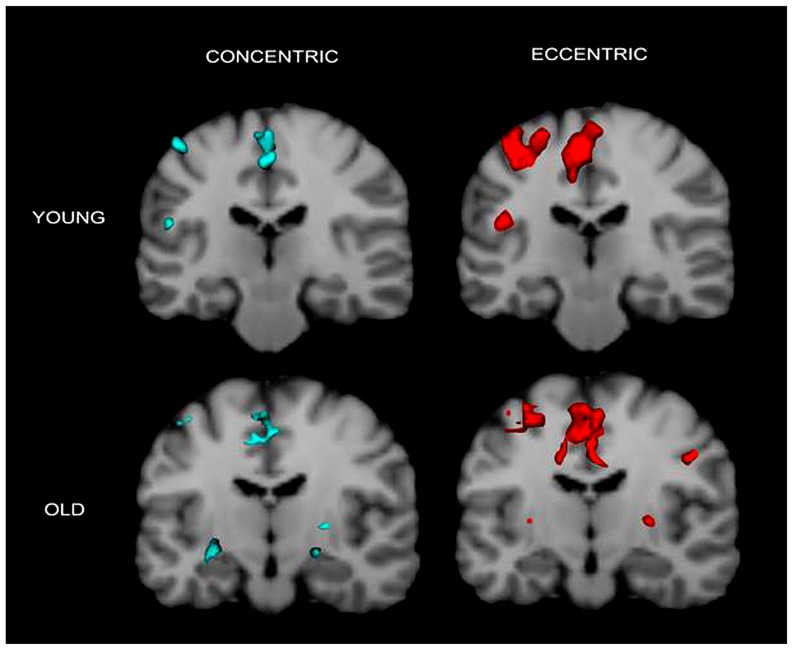
**Cronal view of group activation during the two types of contractions in the two groups.** Again the figure shows that brain activation in young individuals are more localized in the contralateral (left) hemisphere but that in older adults are more distributed in the two hemispheres. Colored spots in the near-top central regions represent activated voxels in SMA and ACC.

The fMRI results revealed activation of a number of motor control-related cortical fields associated with the CC and EC in the left hemisphere contralateral to the performing (right) hand (see **Table 1** and **Figure 2**). For the young group, the activation was almost exclusively in the left hemisphere with the common threshold (*Z* = 3.0) set for activation detection in both groups. For the old group, the ipsilateral (right hemisphere) activation was stronger than young group but was still at a low level compared to the contralateral side (**Figures 2** and **3**). Since our primary interest was to learn potential brain activation difference between controlling CC and EC and the effect of aging on the differences and since the young group had almost no ipsilateral activation, our analysis was mainly focused on activation of the contralateral hemisphere although ipsilateral activation results were also briefly reported and discussed.

**Table 1:** The volume of brain areas activated during CC and EC.

		CC				EC				Volume (mm^3^)	
		*x*	*y*	*z*	*Z*	*x*	*y*	*z*	*Z*	CC (M/SE)	EC (M/SE)
SMA	Young	3	-12	54	3.34	1	-12	54	4.13	533/86	1229/105
	Old	1	-13	52	3.23	2	-11	55	3.72	422/77	1635/167
ACC	Young	4	-6	44	3.41	-2	-10	43	3.27	301/35	457/64
	Old	-5	-10	42	3.26	4	-8	41	3.54	99/22	438/93
Lt. S1	Young	-40	-24	56	3.81	-28	-28	57	3.69	285/52	711/102
	Old	-41	-22	56	3.91	-27	-28	58	3.65	160/20	175/22
Rt. S1	Young	–	–	–	–	–	–	–	–	–	–
	Old	–	–	–	–	38	-28	56	3.05	–	136/22
Lt. PMC	Young	-24	-17	58	3.16	-21	-18	58	3.39	128/26	182/35
	Old	-23	-15	55	3.12	-22	-14	54	3.34	92/17	228/45
Lt. IPL	Young	-46	-33	52	3.45	-32	-38	52	3.21	249/29	278/31
	Old	-47	-32	52	3.56	-32	-37	53	3.61	196/23	265/62
Rt. IPL	Young	–	–	–	–	–	–	–	–	–	–
	Old	–	–	–	–	40	-43	44	3.41	186/23	192/48
Lt. M1	Young	-38	-23	57	3.46	-35	-22	57	3.42	129/35	748/114
	Old	-38	-21	55	3.12	-37	-26	56	3.70	98/18	443/76
Rt. M1	Young	–	–	–	–	–	–	–	–	–	–
	Old	–	–	–	–	20	-26	56	3.29	–	192/26
Rt. CBM	Young	28	-54	-26	3.06	30	-56	-20	3.32	108/7.6	178/14
	Old	33	-67	-21	3.27	32	-60	-20	3.20	188/36	172/23
Med. CBM	Young	–	–	–	–	–	–	–	–	–	–
	Old	8	-58	-28	3.12	16	-68	-24	3.02	90/12	330/27
Lt. Insula	Young	-47	-22	18	3.24	-40	-28	18	3.30	128/22	776/98
	Old	–	–	–	–	–	–	–	–	–	–
Lt. Sup Tem Gyri	Young	–	–	–	–	-46	-36	-14	3.07	–	784/116
	Old	–	–	–	–	–	–	–	–	–	–
Rt. Sup Tem Gyri	Young	–	–	–	–	-48	-45	-16	3.07	–	224/28
	Old	–	–	–	–	–	–	–	–	–	–
Lt. Putamen	Young	–	–	–	–	–	–	–	–	–	–
	Old	-22	3	-6	3.52	-22	-5	9	3.52	264/30	264/20
Rt. Putamen	Young	–	–	–	–	–	–	–	–	–	–
	Old	23	3	-6	3.17	21	-4	7	3.52	128/30	168/20

### ANOVA RESULTS

#### Medial BA6 (SMA)

Activation in the supplementary motor area (SMA) showed a significant main effect for movement type, *F*(1,18) = 98.71, *P* < 0.0001 but not for group, *F*(1,18) = 2.07, *P* = 0.17. There was a significant interaction between the two factors on the fMRI measure, *F*(1,18) = 6.10, *P* = 0.024. *Post hoc* independent *t*-test revealed a significantly greater activation for the elderly than young during EC but not during CC, *t*(18) = 2.139, *P* = 0.046, and *t*(18) = 0.241, *P* = 0.81, respectively (both **Figures 2** and **3** show SMA activation).

#### BA24 (anterior cingulate cortex)

Activation in the anterior cingulate cortex (ACC) showed a significant main effect for movement type, *F*(1,18) = 24.95, *P* < 0.0001 and a boarder-line significant main effect for group, *F*(1,18) = 3.02, *P* = 0.099. There was a significant interaction between the two factors on the fMRI signal, *F*(1,18) = 4.46, *P* = 0.049. *Post hoc* independent *t*-test demonstrated a significantly greater activation for the young than elderly during CC but not during EC, *t*(18) = 4.94, *P* = 0.0001, and *t*(18) = 0.175, *P* = 0.86, respectively (**Figure 3** shows ACC activation in both groups).

#### BA3,1,2 (primary sensory cortex)

Activation in the primary sensory cortex (S1) showed a significant main effect for both group and movement type, *F*(1,18) = 22.07, *P* < 0.0001 and *F*(1,18) = 10.49, *P* < 0.005, respectively. The interaction between the two factors was also significant, *F*(1,18) = 11.01, *P* < 0.005. *Post hoc* paired *t-*tests showed a significantly greater activation during EC than CC in the young group but not in the old group, *t*(10) = 3.70, *P* = 0.004, and *t*(8) = 0.196, *P* = 0.849, respectively.

#### BA4 (primary motor cortex)

Activation in the primary motor cortex (M1) showed a significant main effect for movement type, *F*(1,18) = 53.10, *P* < 0.0001 but not for group, *F*(1,18) = 1.86, *P* = 0.19; nor the interaction effect, *F*(1,18) = 1.71, *P* = 0.21.

#### Lateral BA6 (premotor cortex)

The premotor cortex (PMC) activation exhibited a significant main effect for movement type, *F*(1,18) = 18.83, *P* < 0.0001 but not for group, *F*(1,18) = 2.05, *P* = 0.17; nor the effect oFinteraction, *F*(1,18) = 0.002, *P* = 0.97.

#### BA40 (inferior parietal lobule)

Activation in the inferior parietal lobule (IPL) displayed a significant main effect for movement type, *F*(1,18) = 4.74, *P* < 0.05 but not for group, *F*(1,18) = 0.70, *P* = 0.42; nor the effect oFinteraction, *F*(1,18) = 1.59, *P* = 0.22.

#### Cerebellum

Activation in the cerebellum (CBM) showed a significant main effect for both movement type, *F*(1,18) = 10.57, *P* < 0.01 and group, *F*(1,18) = 58.55, *P* < 0.001. There was no significant interaction between the two factors on the fMRI signal, *F*(1,18) = 2.90, *P* = 0.11 (detailed activation results in all analyzed brain regions are listed in **Table 1**).

### IPSILATERAL ACTIVATION AND ACTIVATION IN YOUNG OR OLD GROUP ONLY

**Table 1** shows that ipsilateral (right) hemisphere activation during EC was only observed in S1, IPL and M1 in the elderly group. Only the old group had activation in both the left and right putamen during both CC and EC. Interestingly, only the young group demonstrated activation during EC in the left and right superior temporal gyri (Sup Tem Gyri) and during both CC and EC in the left insular cortex (**Table 1**).

### EC-TO-CC RATIO RESULTS

The EC-to-CC activation ratio is a measure of relative cortical activation between the two types of contractions in a given brain region in a given subject or subject group. It is striking to see that all the seven regions in which activation occurred during both CC and EC in both groups showed a higher-than-1 ratio (greater activation during EC). In addition, three out of four higher-order cortical fields (SMA, ACC and PMC [except IPL]) exhibited a significantly higher EC-to-CC ratio in the old than the young group, *t*(18) = 4.75, *P* < 0.001; *t*(18) = 4.36, *P* = 0.0001; *t*(18) = 3.96, *P* < 0.001, respectively. The IPL and CBM although exhibited a higher EC-to-CC ratio in the old than the young group, the differences did not reach statistical significance, *t*(18) = 1.15, *P* = 0.263; *t*(18) = 0.41, *P* = 0.687, respectively. In contrast, the two primary motor and sensory cortices (M1 and S1) showed a reversed pattern: the EC-to-CC activation ratio was higher in the young than the old group, *t*(18) = -3.86, *P* < 0.001, and *t*(18) = -5.01, *P* < 0.001, respectively (**Table 2**).

**Table 2:** EC-to-CC ratio in the cortical areas and cerebellum in young and old groups.

	SMA^*^ (M/SE)	ACC^*^ (M/SE)	S1^*^ (M/SE)	PMC^*^ (M/SE)	IPL (M/SE)	M1^*^ (M/SE)	CBM (M/SE)
Young	2.31/0.21	1.52/0.23	2.49/0.29	1.42/0.10	1.12/0.04	5.80/0.24	1.65/0.12
Old	3.87/0.25	4.42/0.67	1.09/0.12	2.48/0.25	1.35/0.23	4.52/0.23

* t-test showing a significant difference in the ratio between young and old groups.

## DISCUSSION

The purpose of this study was to determine if older adults use different central nervous system (CNS) strategies to control concentric (CC, shortening) and eccentric (EC, lengthening) muscle contractions compared with young individuals. Gaining this knowledge is important as CC and EC comprise all of our daily movements. In addition, EC movements are more complex and difficult to control than CC especially for older adults, which poses increased chances of injuries such as falls during walking downstairs (EC of quadriceps) in elders. Knowing the mechanism could potentially help design more targeted therapeutic programs to prevent or reduce chances of such injuries. In general, the activation level (activation volume measured by fMRI) was higher during EC than CC in all cortical regions and cerebellum in both young and old groups, indicating that EC requires greater cortical resources to accomplish the movement. The finding of higher EC-to-CC activation ratio in a number of high-order cortical fields in the old than the young group suggests that the EC is even more difficult to perform in later life compared to early adulthood and this is supported by the poorer movement stability during EC than CC in the old group (Laidlaw et al., 1999).

The general finding of greater cortical activation during EC than CC is consistent with all three previous studies that examined brain signals during the two types of muscle activities (Fang et al., 2001, 2004; Kwon and Park, 2011). The current study provides new evidence supporting the notion that a higher level of complexity of the EC movement poses greater difficulties for one to perform, which demands additional planning, programming, sensorimotor integration and movement execution by the entire central control network. Compared to a CC, an EC is associated with greater movement variability (Laidlaw et al., 1999, 2002), causes more muscle damage especially at a high contraction intensity (Shellock et al., 1991), is possibly accomplished by a different motor unit recruitment strategy (Nardone et al., 1988; Howell et al., 1995), and produces greater force with a given neural signal (EMG; Fang et al., 2001, 2004). These characteristics of EC likely make EC movements more complex and difficult to control compared with CC movements.

Although in principal our fMRI findings are similar to fMRI results of Kwon and Park (2011), there is a substantial contradiction regarding activation in the primary motor cortex (M1). While we observed a larger activation volume in M1 during EC than CC in both groups (see **Tables 1** and **2**), they found that CC was associated with greater M1 activities than EC in young individuals. Kwon and Park (2011) argued that because M1 was traditionally considered to be responsible for movement execution rather than for planning/programming, it was reasonable to see more M1 activity during CC than EC as CC is involved with a higher level of EMG or greater motor unit activities (Fang et al., 2001, 2004). However, recent studies (Lemon, 2008; Kalaska, 2009) report that M1 is not only involved in executing a movement via its direct pathway to motor neuron pool in the spinal cord but also plays an important role in planning the movement. Possible explanations for the different observations regarding M1 activation during EC and CC between the two studies may be attributed to distinct muscles employed for the movements [FDI in our study and wrist extensors in Kwon and Park (2011)] and the load applied to the contractions (we applied 30% maximal load but the other study did not apply an external load). In addition, stronger brain activities found in M1 during EC in our study could be due to M1’s engagement in planning the EC movements to deal with its high degree of movement difficulty. Further research is warranted to better delineate the role of M1 in controlling EC and CC contractions. The following discussion focuses on old and young comparisons especially in the secondary and association cortical areas linked to the EC and CC tasks.

### Activation during EC vs. CC in secondary and association cortices

A major finding of the current study is that although both young and old groups exhibited greater cortical activation during EC than CC, this biased brain activation towards EC was more prominent in the old than the young group especially in the secondary and association cortices. For example, the old group showed a significantly higher EC-to-CC ratio in SMA, PMC and ACC than the young group (the EC-to-CC activation ratio in the IPL was also higher in the old than the young group but did not reach the significance level; **Table 2**). The ANOVA results revealed significantly stronger EC-than-CC activation in the old than the young groups in the SMA and ACC. The role of these higher-order cortical fields in motor control is well described in a standard neuroscience textbook (Part VI / Movement, Kandel et al., 2013). Among these higher-order control centers, the SMA and ACC demonstrated a significantly higher activation level by old than young adults during EC vs. CC shown by both the ANOVA and EC-to-CC ratio analyses, indicating that the two areas play an exceptionally important role in modulating EC movement in later life. It is well known that the SMA is a secondary motor area (consisted of SMA proper and pre-SMA) and is involved in controlling complex and coordinated motor acts (Rizzolatti and Strick, 2013). Given the complex nature of the EC (compared to CC) and its increased level of difficulty (poorer EC movement stability) in late adulthood, it is not surprising to see augmented activity in the SMA during EC in older adults. It has been known that the cingulate association cortex is a part of limbic system that controls emotion, motivation and other cognitive functions. Within the cingulate cortex, however, there exist distinct motor areas in ACC adjacent to the SMA with connections to the M1 and parietal association cortex, and they (SMA and ACC) are considered as an integrated motor control center (termed as medial premotor area) and shared similar functions in motor control (Rizzolatti and Strick, 2013).

In the sole study that compared brain activation patterns between CC and EC in healthy young individuals using fMRI, activation level in the SMA and ACC was seen to be significantly higher during EC than CC (Kwon and Park, 2011). Our study not only confirms this finding in young adulthood but also demonstrates that the biased activation in the SMA and ACC towards EC was even significantly more prominent in older adults than their younger counterparts. We postulate that the SMA and ACC play a special role in modulating EC performance in aging, perhaps by compensating for age-related degenerative adaptations in the motor control network that might have specially deteriorated the network’s ability to control more complex EC movements. The PMC (lateral BA6 consisted of dorsal and ventral PMC, a secondary motor area) showed a significantly higher EC-to-CC activation ratio and the IPL (part of parietal association cortex) also exhibited a clearly higher EC-to-CC ratio (**Table 2**). The motor function of PMC (Rizzolatti and Kalaska, 2013; Rizzolatti and Strick, 2013) and IPL (Fogassi and Luppino, 2005) has been well described and the role of their prominent activation during EC during late life may be similar to that played by the SMA and ACC.

### Cerebellum activation during EC vs. CC

The 2-way ANOVA analysis revealed significant main effects for both movement type and group and no significant effect for interaction which means that the cerebellar activation was greater during EC than CC for both groups and the old group exhibited a higher level of cerebellar activation than the young group. Kwon and Park (2011) reported similar results in young adults. They attributed it (greater cerebellar activity during EC) to the performance of EC movement as early stage learning of the motor skill by their subjects. Although we cannot exclude this possibility (i.e., early learning effect on cerebellar activation), the practice provided before the fMRI scan in our study diminished this likelihood. Thus, we attribute the stronger cerebellar activities observed in EC than CC to its role in dealing with a higher degree of difficulties associated with performing EC than CC movements. The higher activation level in the cerebellum in the old than the young group during both EC and CC movements reflects an increased functional load in the cerebellum for controlling the two types of movements. Previous studies have indicated that the cerebellum is involved in cognitive processes for problem solving (Kim et al., 1994), preparation of motor actions (Coffman et al., 2011), and balance control and movement error correction (Lisberger and Thach, 2013).

### Activation in M1 and S1 during CC and EC

Unlike the secondary and association motor cortices that showed a higher EC-to-CC activation ratio in late adulthood, the primary motor and sensory cortices (M1, S1), however, exhibited a significantly higher such ratio in young than old individuals. It should be noted that the MVC force, movement speed, and movement stability between the young and the old groups did not differ significantly and thus, cannot explain the biased EC-to-CC activation in the M1 and S1in the young group. Instead, while older individuals may need to rely more on the secondary and association cortices to deal with more complex EC movements, the young adults are apt to use the primary motor and sensory areas to handle the more difficult EC. However, the validity of this age-specific brain site for motor planning needs to be further tested by future studies.

### Ipsilateral activation and activation in young or old group only

The above discussion was focused on brain activation in the contralateral (to the performing hand) hemisphere. Besides the major findings in the contralateral side, the fMRI data have shown that ipsilateral (right) hemisphere activation was observed only during EC in M1, S1 and IPL in the elderly group. In addition, only the elderly group had activation in both the left and right putamen in the basal ganglia during both CC and EC. These observations have never been made before and we do not yet know the appropriate explanations. A simple guess would be that these regions played a role in compensating for the worsened ability in the aging control network to manipulate EC movements as these ipsilateral activities were not seen in the young group. Many studies reported increased ipsilateral motor cortex and other region activation and reduced activation laterality during motor performance in healthy aging (e.g., Naccarato et al., 2006). Regarding the observation of bilateral activation in putamen during both CC and EC in the old group, we believe it might be a reflection of heavier use of basal ganglia-cerebellum-cerebral cortex motor control loop during voluntary motor action by older adults. Another interesting finding was that only the young group showed activation during EC in the left and right superior temporal gyri and during both CC and EC in the left insular cortex (**Table 1**). Little is known about the exact role of the temporal and insular cortices in control of CC and EC. In general, the superior temporal cortex is related to perceiving motion (Saygin, 2007) and visuomotor integration (Tanaka et al., 1986). Considering that our subjects performed the motor tasks with visual feedback, it is reasonable to expect activation in the superior temporal cortex. It is unclear though why only the young group showed the activity during the EC action. For activation in the insular cortex, it has been reported that the posterior part of the anterior insular cortex is dedicated for motor control of both upper and lower extremity movements (Mutschler et al., 2009). Again, it is yet to be determined the reason(s) underlying the lack of activation in the insular cortex in late life. Perhaps aging-related structural degeneration such as grey and white matter atrophy in these cortical regions prevented adequate activities in these specific areas.

## SUMMARY

Both young and old individuals demonstrated stronger fMRI-measured activation during EC than CC in almost all cortical areas examined and this biased activation towards EC was more prominent in the old than young groups. Furthermore, all the secondary and association cortices and cerebellum engaged in the two types of muscle contractions exhibited higher EC- than-CC activation in old than young individuals especially in the supplementary motor area and anterior cingulate cortex. On the contrary, the two primary cortices (M1 and S1) showed stronger biased EC activation in young than old age groups. Greater activation in higher-order cortical fields for controlling EC movement in late life may reflect activities in these regions to compensate for impaired ability (perhaps in the primary sensorimotor cortices) to control complex EC movements. All these results associated with aging-related control of EC and CC movements are new and the special roles of the identified cortical fields in modulating EC in late life, especially those at higher levels of the control network justify further delineation by future studies.

## Conflict of Interest Statement

The authors declare that the research was conducted in the absence of any commercial or financial relationships that could be construed as a potential conflict of interest.
